# Contributions to the Chemical and Physiological History of Diabetes Mellitus

**Published:** 1853-04

**Authors:** J. Bryant Smith

**Affiliations:** New York City


// BUFFALO MEDICAL JOURNAL

AND

MONTHLY REVIEW.

VOL. 8. APRIL, 1853. NO. 11.

### ORIGINAL COMMUNICATIONS.

ART. I.—*Contributions to the Chemical and Physiological History of Diabetes Mellitus.* By J. BRYANT SMITH, M. D., of New York City.

This disease has been recognized for a long time as a distinct malady, mention of it being made by Galen, whose attention seems to have been called to it by the extraordinary flow of urine, and not by any peculiarity in the secretion itself, a matter of surprise to us since the sense of taste seems to have been used extensively in diagnosis by the old physicians. The sweet taste of diabetic urine was first noticed by Thomas Willis, and published in 1677, and confirmed by innumerable observers from that time to the present. Ambrosioni obtained sugar from the blood of diabetic patients in large quantities. The identity between this sugar and glucose or grape sugar, was first recognized by Cherreuil, in 1815. Dupuytren and Thenard recognized two species of sugar in the urine, to one of which, having a sweet taste, and possessing the ordinary properties of sugar, they gave the name "sapid," and to the other, wanting these characteristics, they gave the name "insipid." This latter variety has since been shown to be a compound of grape sugar and common salt, a substance obtained by Calloud, in 1825, by evaporating a syrup of diabetic sugar with common salt. Reynoso states that sugar is present in the urine of dogs treated with arsenic, lead, sulphate and carbonate of iron, and that he has detected it also in the urine of patients laboring under tuberculosis, hysteria, chronic bronchitis, pleurisy, asthma and epilepsy.* Michea also detected the same peculiarity in four cases of hysteria, two of epilepsy, and seven of delirium tremens.† The former of these authors also stated that sulphate of quinine, when administered in disease, determines sugar in the urine.

Diabetic urine is light-colored and transparent; of a pale, straw or greenish tint. Its odor is very much like that of an apple chamber, its taste is more or less sweet, and its specific gravity unnaturally high, varying from 1021 to 1050, the healthy average being about 1020. The quantity voided during twenty-four hours, is stated at almost incredible amounts, 40, 70, and even 200 pints, having been reported in this disease. Taking 1040 as the average specific gravity, and ten pints as the average quantity a diabetic patient loses in a day, 15 oz. 7 drams, or more than 1¼ pounds of solid matter per diem. Crystallized diabetic sugar has the composition $C_{24} H_{24} O_{24} + 2 HO$. It may be easily detected in the urine by mixing with it a little sulphate of copper and caustic potash, and boiling the solution, which soon deposits the red suboxide of copper. This test enables us to detect the $\frac{1}{10,000}$ part of grape sugar. (Silliman.)

Messrs. Robin and Verdeil, two French chemists and physiologists, in a new work entitled "Chimie Anatomique et Physiologique," 3 vols., and an atlas published in Paris during the present year, give to this variety of sugar occurring in the disease diabetic mellitus, the name of "Sucre de Foie," or sugar of the liver, a preferable term to diabetic sugar. According to these authors this principle exists in the normal state in the parenchyma of the liver, in the blood of the hepatic veins, in that portion of the vena cava above them, in the blood of the right breast and the pulmonary arteries.

In the young animal we find a trace of sugar in the blood of the pulmonary veins, in the blood of the left breast, in the aorta and its branches, but not in the general veins. During digestion we find it everywhere where it is in the young animal, but in larger quantities, a little being even detected in the nervous system. It is never a constituent of normal bile. It is found from the fourth and fifth month of intra-uterine life to advanced age; it is found in the urine of the fœtus of the cow from four to seven months, and in that of the sheep from six weeks to two months, and also in the liquors aminon and allantoid of these animals.

---

* Comptes Rendus, 1851, vol. 33. † Ibid.

There are some morbid conditions in which we find sugar in those parts of the body which do not normally contain it, and others in which we do not find it where it exists normally.

The disease known as diabetes, is "anatomo-pathologically" characterized by the presence of a great quantity of grape sugar or glucose in the urine. We find it in the saliva, kidneys, serosity of the pericardium and spermatic fluid, as has been shown by Bernard in a dog rendered diabetic. It is not found in the nervous system, nor in the pancreas or spleen. It has been found in the serous fluid of a blister, and also in vomited matter and in the sweat of a patient treated for glucosuria.

There are a number of diseases in which sugar disappears from the liver and is not found there after death, and in diabetic persons during life sugar oftentimes disappears from the urine and even the liver. It diminishes very considerably, or disappears altogether, from animals who become diseased or die from long continued abstinence.

It seems, from these facts, that the duration of sugar in the economy is very great, commencing to appear in the early part of intra-uterine life, and persisting even to the close or near the close of life, although it is liable to be interrupted or entirely absent under certain circumstances.

The quantity of sugar in the liver and blood has never been determined by figures, but we can determine approximately the quantity of tartrate of copper and potassa reduced by a given quantity of the liquid. It is thus that we state the proportion of sugar to be very considerable in man, birds, dogs, horses, rabbits, but much less in reptiles, and entirely absent in the eel and ray. It is also ascertained in this manner that the blood after it leaves the hepatic veins, contains less sugar, and that this decrease gradually continues until it is lost in the pulmonary veins.

In the urine we find the following quantities given by different experimenters:

| | |
|---|---|
| Urine, (Rayer,) . . | 100 parts in 1000. |
| " (Mialhe,) . . | 62 |
| " (Bouchardat,) . | 33 to 134. |
| " (Simon,) . . . | from 25 to 39.80. |
| " (Mialhe of Contour,) | 45 grammes to the litre. |
| " (Reich,) . . . | 43 " in 1000. |
| " (Fouberg,) . . | 32. |
| Blood, (Dr. ——,) . . | .025 grammes in 72 grs. |

Morin, in the French Journal of Pharmacy and Chemistry, 1843, vol. iii, page 354, compares the quantity of urea to that of sugar in 1000 parts of urine with the following results:

| | | Sp. gr. | Sugar grammes. | Urea grammes. |
|---|---|---|---|---|
| November, | 1st Case. | | 21 to 24 | 5.4 |
| December, | | 1025 | 17 | 8.7 |
| June, | | 1026 | 00 | 10.6 |
| December, | | 1034 | 26 | 0.5 |
| November, | 2d Case. | 1040 | 37 | 1.27 |
| January, | | 1027 | 13.5 | 3.64 |
| March, | | 1038 | trace. | 3.32 |
| August, | | 1022 | 0.16 | 7.07 |
| July, | 3d Case. | 1039 | 5.75 | 13.86 |
| August, | | 1037 | 47.00 | 19.31 |
| February, | 4th Case. | 1027 | 45.00 | 0.35 |

Glucose exists in the blood in the liquid state, being dissolved directly in the water. It preserves in the economy the optical properties which it possesses externally; that is, in the urine it turns the polarizing plane to the right; a character which enables us to recognize sugar when it exists in a liquid in the system. "Sugar of the liver" presents in the economy in contact with chemical agents the reactions which are proper to it. Organic substances act on it in the blood, in the liver, in the urine, in the serosity of the pericardium, &c., as out of the system, causing it rapidly to undergo catalysis. In twenty-four hours sugar, which had existed, disappears, and the liquid which had been neutral or alkaline becomes acid. If to urine or blood containing sugar we add a ferment, there will be a decomposition of the grape sugar into alcohol, carbonic acid and water. Sugar combines in the system, as without, with salts; the most of these compounds is that with common salt, having the constitution $C_{24}$ $H_{24}$ $O_{24}$. N. or O. HO. or one equivalent of water in ordinary grape sugar is replaced by one equivalent of chloride of sodium. It is this which we believe to be the compound existing in the disease termed "Diabetes Insipidue." The sugar which exists in the liver is in sufficient quantity to give that organ a little sweetish taste. What are the particular substances or principles which furnish the materials for the formation of sugar in the economy we are unable to say, but M. Bernard has shown that dogs and cats nourished for four, five, or six months entirely on flesh, bones and greasy matters, do not present sugar in the blood which enters into the liver by the vena porta, although the blood in the hepatic veins contains it. Cane sugar ($C_{24}$ $H_{22}$ $O_{22}$) which penetrates by endosmosis into the portal vein, disappears as such in the liver, and passes nto grape sugar, gaining four equivalents of water. It is, perhaps, the same with milk sugar, which gains two equivalents of water ($C_{24} H_{20} O_{20}$) + 2 HO. + 2 HO.=($C_{24} H_{24} O_{24}$.)

It is in the liver that the principal constituents for the formation of sugar are found, and all the causes which influence the circulation in this organ, influence, also, the production of sugar. When the circulation is active here, the quantity of sugar is increased, and vice versa. During digestion the vena porta system is turgescent, and there is present more sugar in the blood whatever be the nature of the food.

Bernard has shown that the formation of sugar is influenced by the nervous system. Not that this system can have any influence on the chemical fact of the combination of the elements hydrogen, carbon, and oxygen in the proportion which constitutes sugar; but the nervous system influences the circulation in the liver, and then follows certain conditions which put into juxtaposition the materials on which depend the formation of glucose.

When we divide the lingual nerve and irritate the periphery of the extremity, we produce no effect; but if we irritate the center we produce effects analogous to those which result from the action of sapid substances on the tongue, that is to say, we have a flow of saliva from the ducts of Warthon and Steno. Thus we have an action transmitted to the nervous centers, and reaction from those on the salivary glands. The pneumogastric nerve is to the lung what the lingual nerve is to the tongue. When we divide this nerve and irritate the inferior extremity, we produce no effect; but if we irritate the superior extremity, there is impression on the nervous center and reflex action on the great sympathetic nerve, which, in its turn, influences the vessels of the liver and there is sugar produced.

This increased production of sugar in the liver which we thus obtain artificially, by irritating the lung, by the administration of chlorine or ether, or by the division of the pneumogastric nerve and irritation of the superior extremity of the nerve, or increasing the reflex action of the medulla oblongata by a wound, is observed in men under some morbid conditions. We find, then, sugar in all the parts of the body of which we have spoken; it is this which characterizes physiologically the disease called "Diabetic Sucrie," a preferable term to "Glucosuria," since we find sugar in all the humors of the body as well as in the urine. The conditions for the formation of glucose from cane sugar, or dextrine in the liver, are only the contact of these substances with the anatomical elements of the liver. In effect many authors have said that the contact of the hepatic parenchyma of a dead animal with cane sugar in solution, was sufficient to cause the sugar to undergo glucose catalysis. Cane sugar which, as such, exists in the vena porta, is passed into glucose in the hepatic veins, and the nervous system acts only to produce the change in the system in a few minutes which requires some hours without this influence.

The crude fecula which is introduced with the intestines comes in contact with nitrogenized bodies and a temperature of about 98° F., there results dextrine catalysis and, perhaps, a small quantity undergoes glucose catalysis, and in these conditions it is carried into the vena porta and thence to the liver, where the glucose catalysis is completed, and the hepatic veins contain sugar.

Finally, we have in the blood the conditions for the destruction of the glucose formed in the normal condition of the system.

Under the influence of fermenting substances, present in the blood, the sugar becomes converted into lactic acid, losing thereof its two equivalents of water and becoming $C_{24}$ $H_{24}$ $O_{24}$; this, reacting on the carbonate, forms lactates of the alkalies present, and as such passes out of the system. If the production of sugar is increased beyond the normal standard, the lactic acid catalysis is no longer able to convert all the sugar into this state, and we find it in the secretions as such.

These views which we have given, alone are founded on the experiments of M. Robin and Verdeil, or M. A. Bernard, of Paris, and are contained in the work of the former authors above cited. M. Bernard's experiments have been published for the most part in the Comptes Rendus de Soc Biol, during the past few years, and will be found highly interesting to those who take an interest in physiological and chemical science as relating to medicine.

Laboratory of the University of Louisville, Ky.

